# Utility of MALDI-TOF MS as a new tool for *Streptococcus pneumoniae* serotyping

**DOI:** 10.1371/journal.pone.0212022

**Published:** 2019-02-12

**Authors:** María Ercibengoa, Marta Alonso, Diego Vicente, Maria Morales, Ernesto Garcia, Jose María Marimón

**Affiliations:** 1 Hospital Universitario Donostia–Instituto de Investigación Sanitaria Biodonostia, San Sebastián, Spain; 2 CIBER de Enfermedades Respiratorias–CIBERES, Madrid, Spain; 3 Preventive Medicine and Health Public Department, University of Basque Country UPV/EHU, San Sebastián, Spain; 4 Departamento de Biotecnología Microbiana y de Plantas, Centro de Investigaciones Biológicas, Consejo Superior de Investigaciones Científicas, Madrid, Spain; Public Health England, UNITED KINGDOM

## Abstract

Nowadays, more than 95 different *Streptococcus pneumoniae* serotypes are known, being less than one third responsible for the majority of severe pneumococcal infections. After the introduction of conjugate vaccines, a change in the epidemiology of the serotypes causing invasive pneumococcal disease has been observed making the surveillance of circulating serotypes especially relevant. Some recent studies have used matrix-assisted laser desorption/ionization time-of-flight mass spectrometry (MALDI-TOF MS) technology to identify the most frequent pneumococcal serotypes that cause invasive disease. The objectives of this study were to evaluate the efficacy of previously described discriminatory peaks determined by MALDI-TOF MS for the identification of serotypes 6B, 19F, 19A and 35B using reference and clinical isolates and to try to identify other discriminatory peaks for serotypes 11A, 19F and 19A using transformed pneumococcal strains. Most of the proposed peaks defined in the literature for the identification of serotypes 6B, 19F, 19A, 35B were not found in the spectra of the 10 reference isolates nor in those of the 60 clinical isolates tested corresponding to these four serotypes. The analysis and comparison of the mass spectra of genetically modified pneumococci (transformed strains) did not allow the establishment of new discriminatory peaks for serotypes 11A, 19F, and 19A. MALDI-TOF MS in the usual range of 2,000 to 20,000 *m*/*z* did not prove to be a valid technique for direct *S*. *pneumoniae* serotyping.

## Introduction

*Streptococcus pneumoniae* serotyping is not currently of the utmost importance for routine clinical practice unlike in the pre-antibiotic era, when it was critical for the patient´s treatment with monospecific antisera [1]. However, serotyping is of great significance from an epidemiological and preventive perspective because it makes possible to define the distribution of serotypes causing invasive pneumococcal disease (IPD) and contributes to updating the composition of pneumococcal vaccines that include the most frequent *S*. *pneumoniae* serotypes causing IPD.

The Quellung reaction has been the “gold standard” technique for *S*. *pneumoniae* serotyping since 1902 [2] in spite of many inconveniences such as being tedious and expensive, including a measure of subjectivity in the interpretation of results and not allowing the analysis of batches of samples [3–4]. Despite these drawbacks, the Quellung reaction continues to be the most frequently used technique for pneumococcal serotyping [5], although other antibody-based alternative methods, such as coagglutination, latex agglutination, ELISA, dot-blot and array techniques have been implemented [6–9]. Additional practices such as counter-immunoelectrophoresis, flow cytometry and immunoblot are cumbersome due to the requirement of special equipment and expertise [10–12].

Since the first works in 2003 on PCR for detecting *S*. *pneumoniae* serotypes on the basis of the nucleotide sequences of capsular genes [13–14], multiplex PCR has become one of the most used typing techniques, allowing the detection of different numbers of serotypes in the same PCR reaction [15]. However, the similitude of the capsular genes among several serotypes makes impossible to differentiate them in a unique reaction so that other alternatives such as DNA sequencing or verifying the serotypes with the Quellung reaction are necessary to differentiate between them; unfortunately, these approaches demand more work and costs [16–17].

Matrix-assisted laser desorption/ionization time-of-flight mass spectrometry (MALDI-TOF MS) has been recently introduced in clinical microbiology laboratories and is considered an excellent method for the identification of most bacterial species. MALDI-TOF MS has also proved to be a reliable tool for other applications, such as antibacterial and antimycotic resistance testing, in addition to some typing applications [18–21]. However, typing applications are extremely new and their use is currently restricted to specialized laboratories [22]. Among these new typing applications, some authors have used MALDI-TOF MS to identify pneumococcal serotypes [23–24].

The main aim of this work was to assess if the discriminatory peaks previously described in the literature for serotypes 6B, 19A, 19F, and 35B [23–24] were present in the spectra of pneumococcal clinical isolates of the same serotypes, regardless of the origin of the sample or the genotype. Another aim was to analyze if the mass spectra obtained by MALDI-TOF MS showed specific discriminatory peaks using three different transformed pneumococci of serotypes 11A, 19F, 19A, and if those peaks were also present in the spectra of clinical isolates of the same serotypes.

## Material and methods

### Clinical isolates

All *S*. *pneumoniae* isolates included in this study were obtained from the frozen (–80C) strain collection available at the Microbiology Department of Donostia University Hospital (Donostia-San Sebastián, Spain). Prior to freezing, all isolates were identified using the optochin sensitivity and bile solubility tests. Pneumococci were serotyped using the Quellung reaction with sera provided by the Statens Serum Institute (Copenhagen, Denmark); serotyping by Quellung was performed in some isolates after determining the capsular type by an in-house designed multiplex PCR [25]. Multi-locus sequence typing (MLST) was performed as previously described [26].

#### Isolates included in the analysis of reported serotype-defining peaks

To evaluate the specificity of the previously described discriminatory peaks [23–24) for serotypes 6B and 19A, 19F, 35B, the mass spectra of 60 clinical isolates previously serotyped 6B (n = 10), 19A (n = 28), 19F (n = 19), and 35B (n = 3), were studied. Seventeen of these isolates had the same sequence type (ST) to those used for defining discriminatory peaks in other studies (Table 1).

**Table 1:** Serotypes and sequence types of the isolates used in this study.

Serotype	Sequence type	Number of strains used in the study
6B	90^1^	4
	386	5
	1624	1
11A	62^1^	4
	3687	1
19F	63	2
	81	1
	88	5
	157	3
	179	3
	271^1^	1
	549	1
	1167	1
	2948	1
	6161	1
19A	193	6
	199^1^	2
	202	3
	276	4
	320^1^	4
	994	2
	1201	1
	2930	1
	3259	2
	4768	1
	198	2
35B	558^1^	2
	4679	1

^1^ Sequence type of the isolates also used in the study of Nakano et al [23]

Besides, 5 reference strains of serotype 6B (ATCC 700670, ATCC 700675 ATCC BAA-342, ATCC BAA-658, ATCC 700903), 3 of serotype 19A (ATCC 700678, ATCC 700904, ATCC 700674), and one each of serotypes 19F (ATCC 700905) and of serotype 35B (Statens Serum Institute serotype 35B) were also included [S1 Table].

#### *S*. *pneumoniae* transformants used in defining serotype-specific peaks

Three encapsulated and one unencapsulated isogenic *S*. *pneumoniae* transformants [27–28] were tested. The strains employed in this study were M11 (unencapsulated derivative of the common laboratory strain R6) and P181, P191 and P242 constructed by transformation of strain M11 with DNA of strains 5086 (serotype 19A), 1064 (serotype 19F) and 2963/13(serotype 11A) respectively [27–28]. Transformants selection was made by enriching encapsulated isolates by successive transfer to C+A medium (CpH8 with 0.08% of bovine seroalbumin) supplemented with 0.5 μl/ml anti-R serum that specifically agglutinates non-encapsulated pneumococci) [28]. Serotyping was performed by Quellung reaction. The above mentioned isolates of serotypes 11A 19F and 19A clinical were also studied to define serotype-specific peaks.

### MALDI-TOF MS studies

Frozen pneumococcal isolates were thawed and cultured onto Trypticase-soy agar with 5% sheep blood plates and incubated at 37C for 20–24 h in a 5% CO_2_ atmosphere. Bacterial extracts for MALDI-TOF MS identification (Bruker, Daltonics, Germany) were obtained by an ethanol–formic acid extraction method to generate high-quality spectra; a 10 μl loopful of bacterial growth was suspended in a mixture of 300 μl of distilled water and 900 μl of ethanol at 96%. The bacterial suspension was centrifuged at 13,000 r.p.m. for 2 min and the supernatant was discarded. The pellet was resuspended in to 50 μl formic acid -water at 70% and left to stand 15 min; subsequently 50 μl of acetonitrile was added. After centrifugation at 13.000 r.p.m for 2 min, 1 μl of the supernatant that was the product of analysis was transferred to the MALDI target plate and overlaid with 1 μl of matrix solution.

[29]. Mass spectra acquisition was performed using a Microflex LT mass spectrometer (Bruker Daltonics, Germany) and the Flex Control software (version 3.4) with default parameter settings. Evaluation of the mass spectra was carried out using the Flex Analysis and MALDI Biotyper 3.1 software and library (version 3.1.66, Bruker Daltonics Germany, with 7.421 entries, last updated March 2018).

Each isolate was analyzed in triplicate and the resulting average measure of each peak (*m*/*z*) was exported to a spreadsheet where a Pearson matrix correlation index was calculated using GraphPad Instat version 3.05 software (GraphPad Software Inc., La Jolla, CA, USA). Cut off intensity was established at 1,000 ua, and a peak tolerance of ±2 Da was admitted as used in other studies [30]. Mass spectra generated by MALDI-TOF were exported in the BTMSP format and visually analyzed using Excel software. Subsequently, the previously discriminatory peaks published by Nakano et al and Pinto et al were evaluated using Microsoft Excel resources”. The same procedure was repeated to try to define discriminatory peaks using the pneumococcal transformants of serotypes 11A, 19F and 19A and its parent unencapsulated isogenic M11 strain. Mass spectrum generated from each isolate was exported and visually analyzed with Microsoft Excel software and the concordance between the peaks of transformants and its parent isogenic unencapsulated strain was evaluated.

## Results

In our study we only included clinical isolates of serotypes 11A, 19F and 19A, because they were the isolates corresponding to the serotype of the available transformed strains, and of serotype 6B that was included in the analysis of Pinto *et al*. [24] and serotype 35B that was also included in the study of Nakano *et al*. [23] together with serotypes 6B, 19F and 19A.

Almost none of the previously described peaks at 6641, 6920, 8424 *m*/*z* identified in > 90% serotype 6B isolates [24] were found among our clinical or reference strains. Only two serotype 6B reference isolates (ATCC BAA658, ATCC 700903) showed the peak at 6270 *m*/*z* described in 50–69% of 6B isolates. Also, most of the discriminatory peaks described by Pinto *et al*. [24] could not be located among clinical isolates. In addition, two serotype 6B ST90 strains showed a peak at 6270 *m*/*z* in their mass spectra, whereas the absence of this peak was highlighted as a characteristic of serotype 6B isolates [24].

Similarly, almost none of the peaks used in the initial and best models generated by ClinProTools of Nakano *et al*. [23] for serotypes 6B, 19A, 19F and 35B were found in the reference strains of the corresponding serotypes studied. Only the peak at 6620 *m*/*z* was found in the 19F reference strain. When analyzing the 60 clinical isolates serotyped with the Quellung reaction as serotypes 6B, 19F, 19A or 35B, only two serotype 6B, two serotype 19A, one serotype 19F and none of the serotype 35B isolates had any of the discriminatory peaks defined for serotyping [23]. In fact, these discriminatory peaks were not only found in the serotypes from which they had been described for but also in the spectra of some isolates of other serotypes. Notably, none of the biomarker peaks used by Pinto *et al*. [24] for serotype 6B corresponded to the peaks described in the models defined by Nakano *et al*. [23] S1 Table.

To attempt finding new discriminatory peaks not previously described in the literature for the identification of serotypes 11A, 19F, 19A, a comparison between the spectra of the unencapsulated M11 strain and of its 3 transformants P181, P191, P242 as well as 40 clinical isolates of the same serotypes 11A (n = 5), 19F (n = 19), 19A (n = 26) was performed. No distinctive peaks could be observed in the spectra of any of the three transformants that had spectral Pearson correlation index of 0.999 between them and with the spectra of the M11 parent strain. Also, no distinctive peaks could be deduced from the spectra of the transformants and the clinical isolates of the same serotype.

## Discussion

This study was focused on analyzing the feasibility of the direct serotyping of *S*. *pneumoniae* using MALDI-TOF MS because of the typing possibilities and the ease and current availability of the MALDI-TOF technology in many clinical microbiology laboratories. The analysis of fingerprinting could have signified a reliable methodology among species within the *Streptococcus mitis* group (SMG) species, which would make routine real-time typing possible. But, despite these advances, an improvement in the database as well as standardization in the protocols employed is needed for the correct characterization of all the species of SMG [31].

The identification of microorganisms by MS is based on the analysis of their protein content, which might create a problem of reproducibility from the theoretical point of view because bacterial protein synthesis can vary depending on the media and culture conditions [32–33]. These variables must remain constant otherwise the mass spectra could be altered [34–35]. In addition, other factors, such as the matrix chosen, the instrument´s calibration, the methodology mass range, the measurements conditions, and even the operator´s skills, can modify the quality of the peaks obtained in the experiment; hence, the importance of technique standardization to improve the reproducibility and comparison of mass spectra [36–37]. All these variables could have influenced the attainment of the expected results because, as shown in this work, clinical isolates and reference strains of serotypes 6B, 19A, 19F and 35B from our region showed peaks different to those previously defined for serotype identification [23–24]. Besides, peaks defined for serotype 6B were also different in both reports. In the work of Pinto *et al*. [24] a validation of the biomarker algorithm with isolates from other regions of the world was proposed but, as observed in our work, the peaks defined did not match with those obtained in the spectra of isolates from our region. However, it does not seem that the absence of discriminatory peaks for serotypes 6B, 19A, 19F and 35B could only be the consequence of a different regional clonal composition, since only two of the serotype-specific discriminatory peaks could be found in two of the 10 reference strains tested.

Previous works on pneumococcal serotyping using MALDI-TOF [23–24] inferred serotypes on the basis of the mass-spectral protein profiles as compared to different peak algorithms in a similar way that serotypes could be inferred form the MLST analysis [38]. Depending on the genetic variability of clinical isolates of a certain serotype in a geographical region, these algorithms will have a better or worse performance (sensitivity and specificity). Of note, MALDI-TOF MS has been also used for the typing of other microorganisms such as *Staphylococcus aureus* or *Enterococcus faecium* without finding specific peaks for a consistent and reliable identification of clonal complexes or sequence types [39].

To date, the molecular identification of pneumococcal serotypes has been done by analyzing the genes encoding the capsular polysaccharide. With the notable exceptions of serotypes 3 and 37, which are synthesized by the synthase pathway [40–41], the polysaccharide capsule of all pneumococcal serotypes is synthetized by what is known as the Wzx/Wzy-dependent pathway [42] and the sequences of the *wzy* gene encoding the polysaccharide polymerase is used for the identification of most serotypes [43]. Hypothetically, differences in the capsular polymerases or synthases could be detected by the MALDI-TOF MS technology, but as demonstrated in this work using transformed strains, specific peaks could not be established for these enzymes. One possible explanation is that the molecular mass of Wzy-type polymerases are over 50 kDa and out of the protein detection range of 2,000 to 20,000 *m*/*z* established by default in the Bruker MALDI-TOF-MS for identifying bacterial species. Perhaps, the use of other technologies such as MALDI-TOF/TOF, capable of detecting proteins of higher molecular mass, could aid in differentiating these enzymes [44]. Another approach could be the use of other mass spectra technology such as electrospray ionization ion trap MS, capable of differentiating carbohydrates, which might allow an accurate discrimination between pneumococcal polysaccharide capsules (serotypes).

This study has some limitations, especially the low number of clinical isolates of each serotype studied that were collected in a small region what could have biased the peaks obtained as a consequence of a low diversity. However, it does not seem that the absence of discriminatory peaks is a direct consequence of the low number of clinical isolates tested because the spectra of the 10 reference strains tested also showed a significant lack of discriminatory peaks.

In this work, no differences in the spectra of parent and transformed *S*. *pneumoniae* strains of three serotypes were found, so that no specific peaks could be assigned to a given serotype. Also, no specific peak could be determined by analyzing clinical *S*. *pneumoniae* isolates of different serotypes. MALDI-TOF MS methodology, in its current form, was not useful for identify *Streptococcus pneumoniae* serotypes currently, because of its low matching with serological and biological molecular techniques.

## Supporting information

S1 Table (TIF)

## References

[1] Podolsky S. The Changing Fate of Pneumonia as a Public Health Concern in 20th-Century America and Beyond. American Journal of Public Health. 2005; 95(12):2144–2154. 10.2105/AJPH.2004.048397 16257952 PMC1449499

[2] Austrian R.The Quellung reaction, a neglected microbiologic technique. Mount Sinai Journal of Medicine. 1976; 43:699–709. 13297

[3] Arai S, Konda T, Wada A, Matsunaga Y, Okabe N, Watanabe H et al Use of Antiserum-Coated Latex Particles for Serotyping *Streptococcus pneumoniae*. Microbiology and Immunology. 2001; 45(2):159–162. 11293482 10.1111/j.1348-0421.2001.tb01284.x

[4] Lafong A, Crothers E. Simple latex agglutination method for typing pneumococci. Journal of Clinical Pathology. 1988; 41(2):230–231. 3350986 10.1136/jcp.41.2.230 PMC1141387

[5] Jauneikaite E, Tocheva A, Jefferies J, Gladstone R, Faust S, Christodoulides M et al Current methods for capsular typing of *Streptococcus pneumoniae*. Journal of Microbiological Methods. 2015; 113:41–49. Journal of Microbiological Methods. 2015; 113:41–49. 10.1016/j.mimet.2015.03.006 25819558

[6] Smart L, Henrichsen J. An Alternative Approach to Typing of *Streptococcus pneumoniae* Strains by Coagglutination. Acta Pathologica Microbiologica Scandinavica Series B: Microbiology. 2009; 94B (1–6):409–413. 10.1111/j.1699-0463.1986.tb03076.x 3565015

[7] Lankinen K, Rintamäki S, Syrjänen R, Kilpi T, Ruutu P, Leinonen M. Type-specific enzyme immunoassay for detection of pneumococcal capsular polysaccharide antigens in nasopharyngeal specimens. Journal of Microbiological Methods. 2004; 56(2):193–199. 14744448 10.1016/j.mimet.2003.10.021

[8] Fenoll A, Jado I, Vicioso D, Casal J. Dot blot assay for the serotyping of pneumococci. Journal of Microbiological 1997; 35:764–766. 10.1128/jcm.35.3.764-766.1997 PMC229668 9041430

[9] Marimon J, Monasterio A, Ercibengoa M, Pascual J, Prieto I, Simón L et al Antibody microarray typing, a novel technique for *Streptococcus pneumoniae* serotyping. Journal of Microbiological Methods. 2010; 80(3):274–280. 10.1016/j.mimet.2010.01.011 20093147

[10] Holliday M. Serotyping of pneumococci by polyvalent counter immunoelectrophoresis (PIE). Journal of Hospital Infection. 1985; 6(1):110–111. 2859314 10.1016/s0195-6701(85)80029-3

[11] Park M, Briles D, Nahm M. A Latex Bead-Based Flow Cytometric Immunoassay Capable of Simultaneous Typing of Multiple Pneumococcal Serotypes (Multibead Assay). Clinical and Vaccine Immunology. 2000;7(3):486–489. 10.1128/cdli.7.3.486-489.2000 PMC95898 10799465

[12] Bronsdon M, O'Brien K, Facklam R, Whitney C, Schwartz B, Carlone G. Immunoblot Method To Detect Streptococcus pneumoniae and Identify Multiple Serotypes from Nasopharyngeal Secretions. Journal of Clinical Microbiology. 2004; 42(4):1596–1600. 10.1128/JCM.42.4.1596-1600.2004 15071010 PMC387534

[13] Brito D, Ramirez M, de Lencastre H. Serotyping *Streptococcus pneumoniae* by Multiplex PCR. Journal of Clinical Microbiology. 2003; 41(6):2378–2384. 10.1128/JCM.41.6.2378-2384.2003 12791852 PMC156574

[14] Lawrence E, Griffiths D, Martin S, George R, Hall L. Evaluation of Semiautomated Multiplex PCR Assay for Determination of *Streptococcus pneumoniae* Serotypes and Serogroups. Journal of Clinical Microbiology. 2003;41(2):601–607. 10.1128/JCM.41.2.601-607.2003 12574253 PMC149661

[15] Siira L, Kaijalainen T, Lambertsen L, Nahm M, Toropainen M, Virolainen A. From Quellung to Multiplex PCR, and Back When Needed, in Pneumococcal Serotyping. Journal of Clinical Microbiology. 2012; 50(8):2727–2731. 10.1128/JCM.00689-12 22692742 PMC3421528

[16] Leung M, Bryson K, Freystatter K, Pichon B, Edwards G, Charalambous B et al Sequetyping: Serotyping *Streptococcus pneumoniae* by a Single PCR Sequencing Strategy. Journal of Clinical Microbiology. 2012; 50(7):2419–2427. 10.1128/JCM.06384-11 22553238 PMC3405617

[17] Varghese R, Jayaraman R, Veeraraghavan B. Current challenges in the accurate identification of *Streptococcus pneumoniae* and its serogroups/serotypes in the vaccine era. Journal of Microbiological Methods. 2017; 141:48–54. 10.1016/j.mimet.2017.07.015 28780272

[18] Wolters M, Rohde H, Maier T, Belmar-Campos C, Franke G, Scherpe S et al MALDI-TOF MS fingerprinting allows for discrimination of major methicillin-resistant Staphylococcus aureus lineages. International Journal of Medical Microbiology. 2011; 301(1):64–68. 10.1016/j.ijmm.2010.06.002 20728405

[19] Griffin P, Price G, Schooneveldt J, Schlebusch S, Tilse M, Urbanski T et al Use of Matrix-Assisted Laser Desorption Ionization-Time of Flight Mass Spectrometry To Identify Vancomycin-Resistant Enterococci and Investigate the Epidemiology of an Outbreak. Journal of Clinical Microbiology. 2012; 50(9):2918–2931. 10.1128/JCM.01000-12 22740710 PMC3421795

[20] Bader O. MALDI-TOF-MS-based species identification and typing approaches in medical mycology. PROTEOMICS. 2013; 13(5):788–799. 10.1002/pmic.201200468 23281257

[21] Qian J, Cutler J, Cole R, Cai Y. MALDI-TOF mass signatures for differentiation of yeast species, strain grouping and monitoring of morphogenesis markers. Analytical and Bioanalytical Chemistry. 2008; 392(3):439–449. 10.1007/s00216-008-2288-1 18690424

[22] Dingle T, Butler-Wu S. MALDI-TOF Mass Spectrometry for Microorganism Identification. Clinics in Laboratory Medicine. 2013; 33(3):589–609. 10.1016/j.cll.2013.03.001 23931840

[23] Nakano S, Matsumura Y, Ito Y, Fujisawa T, Chang B, Suga S et al Development and evaluation of MALDI-TOF MS-based serotyping for *Streptococcus pneumoniae*. European Journal of Clinical Microbiology & Infectious Diseases. 2015; 34(11):2191–2198. 26282790 10.1007/s10096-015-2468-9

[24] Pinto T, Costa N, Castro L, Ribeiro R, Botelho A, Neves F et al Potential of MALDI-TOF MS as an alternative approach for capsular typing Streptococcus pneumoniae isolates. Scientific Reports. 2017;7(1). 10.1038/srep45572 PMC5368646 28349999

[25] Marimón J, Ercibengoa M, Santacatterina E, Alonso M, Pérez-Trallero E. Single-Step Multiplex PCR Assay for Determining 92 Pneumococcal Serotypes. Journal of Clinical Microbiology. 2016; 54(8):2197–2200. 10.1128/JCM.01156-16 27280423 PMC4963499

[26] Enright M, Spratt B. A multilocus sequence typing scheme for *Streptococcus pneumoniae*: identification of clones associated with serious invasive disease. Microbiology. 1998; 144(11):3049–3060. 9846740 10.1099/00221287-144-11-3049

[27] Domenech M, Damián D, Ardanuy C, Liñares J, Fenoll A, García E. Emerging, Non-PCV13 Serotypes 11A and 35B of Streptococcus pneumoniae Show High Potential for Biofilm Formation In Vitro. PLOS ONE. 2015; 10(4):e0125636 10.1371/journal.pone.0125636 25927917 PMC4415931

[28] Domenech M, Araújo-Bazán L, García E, Moscoso M. In vitrobiofilm formation by *Streptococcus pneumoniae* as a predictor of post-vaccination emerging serotypes colonizing the human nasopharynx. Environmental Microbiology. 2014;16(4):1193–1201. 10.1111/1462-2920.12370 24373136

[29] MALDI Biotarget 48. Instructions for Use. Disposable MALDI targets for microorganism identification. Revision 1 (December 2011)—PDF [Internet]. Docplayer.net. 2018 [cited 12 July 2018]. Available from: http://docplayer.net/24726764-Maldi-biotarget-48-instructions-for-use-disposable-maldi-targets-for-microorganism-identification-revision-1-december-2011.html Bruker. 2011. Instructions for use MALDI Biotarget 48. https://www.criver.com/sites/default/files/resources/InstructionsforUsingMALDIBiotarget48.pdf.

[30] Månsson V, Gilsdorf J, Kahlmeter G, Kilian M, Kroll J, Riesbeck K et al Capsule Typing of Haemophilus influenzae by Matrix-Assisted Laser Desorption/Ionization Time-of-Flight Mass Spectrometry1. Emerging Infectious Diseases. 2018;24(3):443–452. 10.3201/eid2403.170459 29460728 PMC5823330

[31] Ercibengoa M. MALDI-TOF MS Breakpoint Limitations for Streptococcus mitis group species typing. Clinical Laboratory. 2017; 63:1935–1938.

[32] Goldstein J, Zhang L, Borror C, Rago J, Sandrin T. Culture conditions and sample preparation methods affect spectrum quality and reproducibility during profiling of *Staphylococcus aureus* with matrix-assisted laser desorption/ionization time-of-flight mass spectrometry. Letters in Applied Microbiology. 2013; 57(2):144–150. 10.1111/lam.12092 23617594

[33] Balážová T, Makovcová J, Šedo O, Slaný M, Faldyna M, Zdráhal Z. The influence of culture conditions on the identification of Mycobacterium species by MALDI-TOF MS profiling. FEMS Microbiology Letters. 2014; 353(1):77–84. 10.1111/1574-6968.12408 24571790

[34] Pavlovic M. Application of MALDI-TOF MS for the Identification of Food Borne Bacteria. The Open Microbiology Journal. 2013; 7(1):135–141. 24358065 10.2174/1874285801307010135 PMC3866695

[35] Ueda O, Tanaka S, Nagasawa Z, Hanaki H, Shobuike T, Miyamoto H. Development of a novel matrix-assisted laser desorption/ionization time-of-flight mass spectrum (MALDI-TOF-MS)-based typing method to identify meticillin-resistant Staphylococcus aureus clones. Journal of Hospital Infection. 2015; 90(2):147–155. 10.1016/j.jhin.2014.11.025 25922338

[36] Keys C, Dare D, Sutton H, Wells G, Lunt M, McKenna T et al Compilation of a MALDI-TOF mass spectral database for the rapid screening and characterisation of bacteria implicated in human infectious diseases. Infection, Genetics and Evolution. 2004; 4(3):221–242. 10.1016/j.meegid.2004.02.004 15450202

[37] Hettick J, Kashon M, Simpson J, Siegel P, Mazurek G, Weissman D. Proteomic Profiling of Intact Mycobacteria by Matrix-Assisted Laser Desorption/Ionization Time-of-Flight Mass Spectrometry. Analytical Chemistry. 2004; 76(19):5769–5776. 10.1021/ac049410m 15456297

[38] Williamson Y, Moura H, Woolfitt A, Pirkle J, Barr J, Carvalho M et al Differentiation of *Streptococcus pneumoniae* Conjunctivitis Outbreak Isolates by Matrix-Assisted Laser Desorption Ionization-Time of Flight Mass Spectrometry. Applied and Environmental Microbiology. 2008; 74(19):5891–5897. 10.1128/AEM.00791-08 18708515 PMC2565961

[39] Lasch P, Fleige C, Stämmler M, Layer F, Nübel U, Witte W et al Insufficient discriminatory power of MALDI-TOF mass spectrometry for typing of *Enterococcus faecium and Staphylococcus aureus isolates*. Journal of Microbiological Methods. 2014; 100:58–69. 10.1016/j.mimet.2014.02.015 24614010

[40] Arrecubieta C. Type 3-specific synthase of Streptococcus pneumoniae (Cap3B) directs type 3 polysaccharide biosynthesis in Escherichia coli and in pneumococcal strains of different serotypes. Journal of Experimental Medicine. 1996; 184(2):449–455. 8760798 10.1084/jem.184.2.449 PMC2192726

[41] Llull D, Muñoz R, López R, García E. A Single Gene (tts) located outside the cap Locus Directs the Formation of Streptococcus pneumoniae Type 37 Capsular Polysaccharide. The Journal of Experimental Medicine. 1999; 190(2):241–252. 10432287 10.1084/jem.190.2.241 PMC2195575

[42] Bentley S, Aanensen D, Mavroidi A, Saunders D, Rabbinowitsch E, Collins M et al Genetic Analysis of the Capsular Biosynthetic Locus from All 90 Pneumococcal Serotypes. PLoS Genetics. 2006; 2(3):e31 10.1371/journal.pgen.0020031 16532061 PMC1391919

[43] Varvio S, Auranen K, Arjas E, Mäkelä P. Evolution of the Capsular Regulatory Genes in *Streptococcus pneumoniae*. The Journal of Infectious Diseases. 2009; 200(7):1144–1151. 10.1086/605651 19705970

[44] Shah H N., Culak R A. Changes in the Matrix Markedly Enhance the Resolution and Accurate Identification of Human Pathogens by MALDI-TOF MS. Journal of Analytical & Bioanalytical Techniques. 2014; s2 (01).

