# Supplementary figures and images for: Utility of MALDI-TOF MS as a new tool for Streptococcus pneumoniae serotyping
Source: PLoS One. 2019 Feb 12;14(2):e0212022. doi: 10.1371/journal.pone.0212022 (PMC6372175; doi:10.1371/journal.pone.0212022)

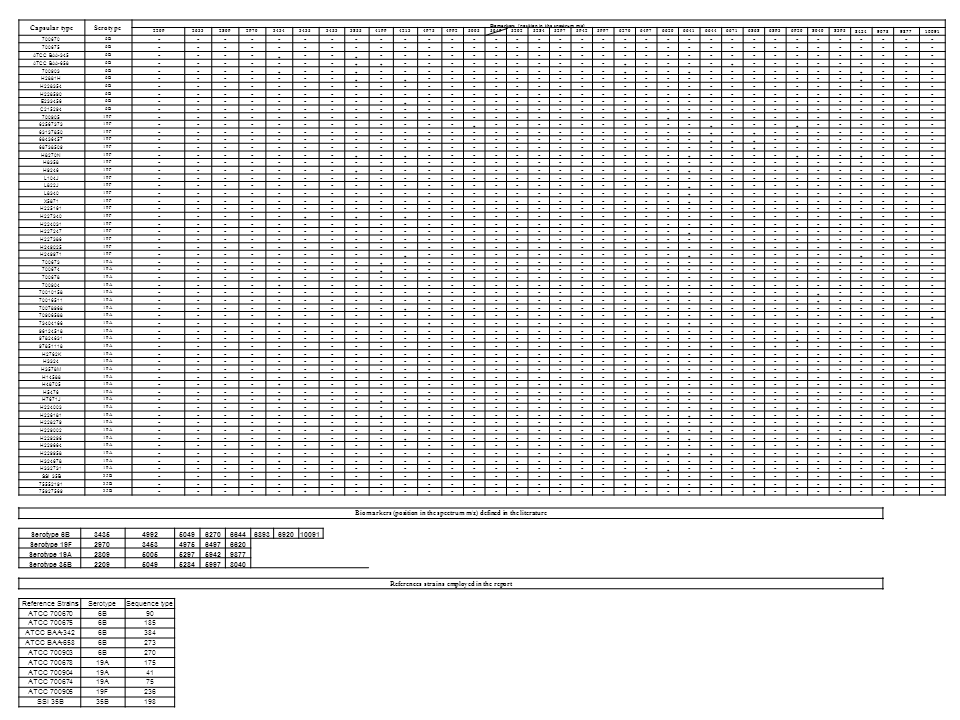

Supplement: S1 Table — (TIF) [file pone.0212022.s001.tif]
